# Prospective Association between Dietary Fiber Intake and Breast Cancer Risk

**DOI:** 10.1371/journal.pone.0079718

**Published:** 2013-11-14

**Authors:** Mélanie Deschasaux, Laurent Zelek, Camille Pouchieu, Mathilde His, Serge Hercberg, Pilar Galan, Paule Latino-Martel, Mathilde Touvier

**Affiliations:** 1 Nutritional Epidemiology Research Team, Sorbonne Paris Cité Research Center, Inserm (U557), Inra (U1125), Cnam, Paris 13 University, SMBH Paris 13, Bobigny, France; 2 Oncology Department, Avicenne Hospital, Bobigny, France; 3 Public Health Department, Avicenne Hospital, Bobigny, France; Wayne State University School of Medicine, United States of America

## Abstract

**Background:**

Mechanistic hypotheses suggest a potential effect of dietary fiber on breast carcinogenesis through the modulation of insulin-like growth factor bioactivity, estrogen metabolism and inflammation. An association between dietary fiber intake and breast cancer risk has been suggested in epidemiological studies but remains inconclusive. In particular, data is lacking regarding the different types of dietary fibers.

**Objective:**

The objective was to investigate the prospective relationship between dietary fiber intake and breast cancer risk, taking into account different types of dietary fiber (overall, insoluble, soluble and from different food sources: cereals, vegetables, fruits and legumes).

**Design:**

4684 women from the SU.VI.MAX cohort were included in this analysis as they completed at least three 24h-dietary records within the first two years of follow-up. Among them, 167 incident invasive breast cancers were diagnosed during a median follow-up of 12.6 years (between 1994 and 2007). The associations between quartiles of dietary fiber intake and breast cancer risk were characterized using multivariate Cox proportional hazards models.

**Results:**

Total fiber intake was not associated with breast cancer risk (HR_Quartile4vs.Quartile1_ = 1.29 (95%CI 0.66–2.50), *P*-trend = 0.5), nor was fiber intake from cereals (*P*-trend = 0.1), fruits (*P*-trend = 0.9) and legumes (*P*-trend = 0.3). In contrast, vegetable fiber intake was related to a decreased risk of breast cancer (HR_Q4vs.Q1_ = 0.50 (0.29-0.88), *P*-trend = 0.03). Overall vegetable intake (in g/day) was not associated with breast cancer risk (*P*-trend = 0.2).

**Conclusion:**

This prospective study suggests that vegetable fiber intake may contribute to reduce breast cancer risk, in line with experimental mechanistic data.

## Introduction

Several mechanisms are involved in breast cancer development. First, insulin-resistance and its consequences such as higher insulin-like growth factor (IGFs) bioactivity [1], [2] or lower sex-hormone binding globulin (SHBG) [3] concentration have been associated with increased breast cancer risk in experimental [3], [4] and epidemiological [5]–[7] studies. Second, epidemiological data suggest a relationship between breast cancer risk and increased circulating estrogens [6], [8], [9]. Finally, inflammation process may play a role in breast carcinogenesis, as shown in experimental [3], [4], [10], [11] and epidemiological [12], [13] studies. Mechanistic hypotheses support a role for dietary fiber in the prevention of breast cancer through a reduction of IGFs bioactivity, notably by increasing insulin-like growth factor binding protein 3 (IGFBP3) concentration [14], [15]; an influence on steroid hormone concentrations by decreasing circulating estrogens [16] and upregulating SHBG concentrations [17] and a reduction of inflammation, thanks to the production of short-chain fatty acid (SCFA) by colonic fermentation [18]–[21].

However, epidemiological evidence is lacking. In the Continuous Update Project of the World Cancer Research Fund (WCRF) / American Institute for Cancer Research (AICR) published in 2010 [22], the expert committee stated that the epidemiological evidence regarding the association between dietary fiber intake and breast cancer risk was insufficient to conclude. Since then, two meta-analyses of prospective studies have been published, suggesting a decreased breast cancer risk associated with dietary fiber intake [23], [24]. After these two meta-analyses, one prospective study, based on the EPIC cohort, has been published with similar results [25]. However, questions remain regarding the type of dietary fiber involved in this association. Different types of dietary fiber could have differential effects on breast cancer development as the definition of “dietary fiber” refers to a large category of molecules with potentially different properties and physiological effects [26]. So far, epidemiological data remain limited and contrasted: one meta-analysis reported inverse association between soluble fiber intake and breast cancer risk, but no association with insoluble fiber intake nor with fiber intake from cereals, vegetables, fruits and legumes [24], whereas the recent large prospective EPIC study observed an inverse association between vegetable fiber intake and breast cancer risk [25]. Thus, new prospective studies considering different types of dietary fibers are needed to further investigate the relationship between dietary fiber intake and breast cancer risk.

Therefore, our objective was to prospectively investigate the association between different types of dietary fiber (overall, insoluble, soluble and from different food sources: cereals, vegetables, fruits and legumes) and breast cancer risk.

## Subjects and Methods

### Ethics Statement

The SU.VI.MAX study was conducted according to the Declaration of Helsinki guidelines and was approved by the Ethics Committee for Studies with Human Subjects of Paris-Cochin Hospital (CCPPRB n° 706 and n° 2364, respectively) and the Commission Nationale de l’Informatique et des Libertés (CNIL n° 334641 and n° 907094, respectively). Written informed consent was obtained from all participants.

### Subjects

The SU.VI.MAX study (SUpplementation en VItamines et MInéraux AntioXydants) was at first designed as a randomized, double-blind, placebo-controlled primary prevention trial (Trial Registration clinicaltrials.gov Identifier: NCT00272428) aiming to assess the effect of a daily supplementation with nutritional doses of antioxidants on the incidence of cardiovascular diseases and cancers [27]. 13,017 subjects were recruited in 1994–1995 for an 8-y intervention study and were then followed for health events until September 2007.

### Baseline data collection

At enrollment, self-administered questionnaires related to socio-demographics, smoking status, physical activity and family history of breast cancer were filled-in by all participants. Anthropometric measures were performed by the study’s nurses and physicians during a medical examination.

During the trial period (1994–2002), participants were invited to complete a 24h-dietary record every two months. These records were randomly distributed between weeks and week-ends and over seasons to take into account intra-individual variability. In order to be consistent with a prospective design, only dietary records from the first two years of follow-up were used in the present study. Completion was made through the Minitel Telematic Network, a French telephone-based terminal equivalent to an Internet prototype. Portion sizes were assessed thanks to a validated picture booklet [28] and the amounts consumed from composite dishes were estimated using French recipes validated by food and nutrition professionals. The mean daily energy, alcohol, and nutrient intakes were estimated using a published French food composition table [29]. Total dietary fiber and soluble fiber contents were obtained using the Association of Official Analytical Chemists method for total dietary fiber (AOAC 985.29) with modifications for soluble fiber measurement [30]. Dietary fiber intakes in the SU.VI.MAX study were previously described [31].

### Case ascertainment

Health events occurring during the follow-up were self-reported by participants. Medical data were then gathered through participants, physicians, and/or hospitals and reviewed by an independent physician expert committee. Pathological reports were used to validate the cases and to extract cancer characteristics (histological type, estrogen and progesterone receptors, tumor size, number of nodes, cancer grade). Cases were classified using the International Chronic Diseases Classification, 10th Revision, Clinical Modification (ICD-10) [32]. All first incident invasive primary breast cancers were considered as cases in this study.

### Statistical analyses

From the 7876 female participants in the SU.VI.MAX study, we excluded 120 women who reported a cancer diagnosis before the start of the follow-up. Among the remaining subjects, 4684 provided at least three valid 24h-dietary records within the first two years of follow-up and thus remained available for analysis. For overall breast cancer analysis, women contributed person-time until the date of diagnosis of breast cancer, the date of last completed questionnaire, the date of death, or September 2007, whichever occurred first. For analyses stratified by menopausal status, women contributed person-time until their date of menopause for premenopausal breast cancer analysis or from their date of menopause for postmenopausal breast cancer analysis. Women who reported a cancer other than breast cancer (N = 164) or a non-invasive breast cancer (N = 23) during the study period were included and censored at the date of diagnosis (except basal cell skin carcinoma, not considered as cancer). Nutrient intakes were estimated by the average intake calculated from all dietary records for each woman.

Baseline characteristics of participants were compared between quartiles of total dietary fiber intake, using Chi-square tests or Fisher tests where appropriate. Hazards ratios (HRs) and 95% Confidence Intervals (CIs)), obtained from Cox proportional hazards models with age as the primary time variable, were used to characterize the association between quartiles of dietary fiber intake and incident breast cancer. We verified that the assumptions of proportionality were satisfied through examination of the log–log (survival) versus log–time plots. Different categories of dietary fibers were tested: according to their chemical properties (soluble and insoluble fibers) and according to their food sources (cereal, vegetable, fruit and legume fibers). Tests for linear trend were performed using the ordinal score on quartiles of fiber intake. Multivariate models were adjusted for intervention group of the initial SU.VI.MAX trial (yes/no), smoking status (never, former or current), educational level (primary, secondary or university), physical activity (irregular, <1h/d or ≥1h/d walking or equivalent), height (continuous), body mass index (BMI; continuous), without-alcohol energy intake (continuous), alcohol intake (continuous), total fat intake (continuous), number of dietary records (continuous), family history of breast cancer (yes/no), number of children (continuous), menopausal status at baseline (yes/no) and use of hormonal treatment for menopause (HTM) at baseline (yes/no). Since a high fiber intake might reflect an overall healthy diet and since we aimed at disentangling the potential effect of dietary fiber from the effect of other components of a healthy diet, we adjusted the multivariate models for a healthy dietary pattern. This healthy pattern was extracted by principal component analysis, using the SAS “proc factor” procedure with the ‘‘Varimax’’ option, from mean intakes across all 24-h records collected during the first 2 years of the study, for 31 food groups. For interpreting the data, we considered food groups with a factor loading under –0.2 or over 0.2. The factor that was strongly correlated with vegetables, fruits and seafood intakes was considered as “healthy pattern”. A score characterizing the adequacy of each woman with this pattern was calculated by summing the intakes of all food groups weighted by the food group factor loadings [33], [34]. Specific models were computed for ductal breast cancers, estrogen receptor positive (ER+) or progesterone receptor positive (PR+) breast cancers and postmenopausal breast cancers. Lobular and other histological types of breast cancers, as well as ER and/or PR negative breast cancers and premenopausal breast cancers could not be tested due to an insufficient number of cases. For these analyses, breast cancer cases with different characteristics from the studied ones were excluded. All tests were two-sided and P<0.05 was considered statistically significant. SAS version 9.3 (SAS Institute, Cary, NC) was used for analyses.

## Results

During a median follow-up of 12.6 years (52,944 person-years), 167 women developed a first primary invasive breast cancer with a mean age at diagnosis of 55.8 years. **TABLE 1** presents the characteristics of the subjects according to quartiles of total dietary fiber intake. Women in the upper quartile tended to be older, leaner, taller, non-smoker, to practice more physical activity and to have a higher education degree. The average fiber intake was 17.2 g/day (SD = 5.9). Only 8.6% of the study population reached the 25 g/day minimal French recommendation [35]. Main contributors to dietary fiber intake were cereals (36.3%), vegetables (23.8%), fruits (21.9%) and legumes (5.7%). Pearson correlation coefficients of total dietary fiber intake with cereal, fruit, vegetable and legume fiber intakes were respectively 0.69, 0.66, 0.60 and 0.37.

**Table 1:** Baseline characteristics of the women (N = 4684) according to quartiles of total fiber intake, SU.VI.MAX cohort, France, 1994–2007.

	Q1 (n = 1171)		Q2 (n = 1171)		Q3 (n = 1171)		Q4 (n = 1171)		*P* 1
Age (years)	46.6	±6.3	46.6	±6.7	47.1	±6.6	47.6	±6.7	*<0.0001*
BMI (kg/m^2^)	23.4	±4.0	23.0	±3.8	23.0	±3.5	23.0	±3.6	*0.005*
*≥25 kg/m^2^*	306	(26.1)	242	(20.7)	246	(21.0)	246	(21.0)	*0.003*
Height (cm)	161	±6.0	161	±5.9	162	±5.7	163	±5.9	*<0.0001*
Intervention group (yes)	552	(47.1)	569	(48.6)	602	(51.4)	594	(50.7)	*0.1*
Smoking status									*<0.0001*
*Never*	594	(50.7)	671	(57.3)	700	(59.8)	739	(63.1)	
*Former*	329	(28.1)	327	(27.9)	353	(30.2)	338	(28.9)	
*Current*	248	(21.2)	173	(14.8)	118	(10.1)	94	(8.0)	
Physical activity									*0.0001*
*Irregular*	347	(29.6)	312	(26.6)	295	(25.2)	245	(20.9)	
*<1h/d walking or equivalent*	367	(31.3)	406	(34.7)	428	(36.6)	435	(37.2)	
*≥1h/d walking or equivalent*	457	(39.0)	453	(38.7)	448	(38.3)	491	(41.9)	
Educational level									*<0.0001*
*Primary*	271	(23.1)	196	(16.7)	204	(17.4)	181	(15.5)	
*Secondary*	454	(38.8)	469	(40.1)	466	(39.8)	456	(38.9)	
*University*	446	(38.1)	506	(43.2)	501	(42.8)	534	(45.6)	
Family history of breast cancer^2^ (yes, %)	104	(8.9)	108	(9.2)	110	(9.4)	85	(7.3)	*0.2*
Number of children	2	±1.1	2	±1.1	2	±1.1	2	±1.2	*0.8*
Menopausal status at baseline (yes, %)	337	(28.8)	341	(29.1)	358	(30.6)	377	(32.2)	*0.3*
Age at menopause (years)	51.0	±4.7	51.1	±4.3	50.9	±4.2	51.2	±3.9	*0.4*
Use of HTM at baseline (yes,%)	317	(27.1)	330	(28.2)	375	(32.0)	366	(31.3)	*0.02*
Energy intake (kcal/d)	1438.3	±367.6	1746.2	±345.5	1934.7	±363.9	2188.9	±446.3	*<0.0001*
Alcohol intake (g/d)	11.9	±15.8	11.3	±13.0	10.9	±12.7	8.8	±10.8	*0.006*
Total fat intake (g/d)	63.9	±19.9	76.8	±18.9	84.2	±19.9	93.9	±25.2	*<0.0001*
Total dietary fiber intake (g/d)	10.7	±2.0	15.0	±0.9	18.4	±1.1	24.9	±4.9	*<0.0001*
*Insoluble fiber (g/d)*	8.4	±1.7	11.9	±0.9	14.6	±1.0	19.9	±4.1	*<0.0001*
*Soluble fiber (g/d)*	2.3	±0.5	3.1	±0.5	3.8	±0.6	5.0	±1.2	*<0.0001*
*Cereal fiber (g/d)*	4.1	±1.4	5.6	±1.7	6.7	±2.0	8.7	±3.3	*<0.0001*
*Vegetable fiber (g/d)*	2.7	±1.2	3.6	±1.4	4.4	±1.6	5.7	±2.2	*<0.0001*
*Fruit fiber (g/d)*	2.0	±1.3	3.2	±1.5	4.1	±1.8	5.8	±2.9	*<0.0001*
*Legume fiber (g/d)* 3	0.5	±0.7	0.7	±0.9	1.1	±1.2	1.7	±2.0	*<0.0001*
Score of overall healthy dietary pattern	–0.6	±0.8	–0.2	±0.8	0.1	±0.8	0.7	±1.0	*<0.0001*

BMI body mass index; HTM hormonal treatment for menopause; Q Quartile.

Values are mean ±SD for all variables except for BMI≥25 kg/m^2^, intervention group, smoking status, physical activity, educational level, family history of breast cancer, menopausal status at baseline and use of HTM at baseline for which they are N, %.

1 Chi-square tests or Fisher tests as appropriate. Data for dietary variables were log-transformed to improve normality. All statistical tests were 2-sided.

2 Among first degree relatives.

3 Including fiber from soya and soya products.

Associations between dietary fiber intake and breast cancer risk are summarized in **TABLE 2**. Total fiber intake was not associated with breast cancer risk (HR_Quartile4vs.Quartile1_ = 1.29 (95%CI 0.66–2.50), *P*-trend = 0.5), nor was fiber intake from cereals (*P*-trend = 0.1), fruits (*P*-trend = 0.9) and legumes (*P*-trend = 0.3). In contrast, vegetable fiber intake was associated with a decreased breast cancer risk (HR_Q4vs.Q1_ = 0.50 (0.29-0.88), *P*-trend = 0.028). We verified that this result was observed for postmenopausal breast cancers (n = 116, HR_Q4vs.Q1_ = 0.50 (0.26–0.97), *P*-trend = 0.03), ductal breast cancers (n = 119, HR_Q4vs.Q1_ = 0.42 (0.21–0.83), *P*-trend = 0.02), ER+ (n = 113, HR_Q4vs.Q1_ = 0.37 (0.18–0.76), *P*-trend = 0.02) and PR+ (n = 86, HR_Q4vs.Q1_ = 0.36 (0.16–0.81), *P*-trend = 0.046) breast cancers (data not tabulated).

**Table 2:** Associations between quartiles of dietary fiber intake and breast cancer risk from multivariate Cox proportional hazards models^1^, SU.VI.MAX cohort, France, 1994–2007 (167 cases /4684 women).

	Q1	Q2		Q3		Q4		
Dietary fiber	HR	HR	95% CI	HR	95%CI	HR	95%CI	*P for trend*
Total fiber	1	1.19	0.73–1.93	1.18	0.69–2.03	1.29	0.66–2.50	*0.5*
Insoluble fiber	1	1.22	0.75–1.99	1.29	0.75–2.22	1.32	0.68–2.57	*0.4*
Soluble fiber	1	1.12	0.70–1.81	1.12	0.67–1.87	1.22	0.67–2.22	*0.6*
Cereal fiber	1	1.05	0.64–1.72	1.44	0.88–2.38	1.43	0.81–2.53	*0.1*
Vegetable fiber	1	0.83	0.54–1.28	0.83	0.53–1.30	0.50	0.29–0.88	*0.028*
Fruit fiber	1	0.92	0.58–1.45	0.86	0.54–1.39	1.07	0.64–1.79	*0.9*
Legume fiber	1	1.63	1.03–2.59	1.25	0.77–2.04	1.44	0.90–2.31	*0.3*

HR Hazard Ratio; CI Confidence Interval; Q Quartile.

1 Adjusted for age (time scale), intervention group, smoking status, educational level, physical activity, height, BMI, number of dietary records, without-alcohol energy intake, alcohol intake, total fat intake, overall healthy dietary pattern, family history of breast cancer, menopausal status at baseline, use of HTM at baseline and number of children.

Cut-offs (g/d) for quartiles of dietary fiber intakes were 13.3/16.6/20.3 for total fiber, 10.5/13.2/16.2 for insoluble fiber, 2.7/3.4/4.2 for soluble fiber, 4.4/5.9/7.7 for cereal fiber, 2.7/3.8/5.2 for vegetable fiber, 2.1/3.4/5 for fruit fiber and 0.05/0.5/1.4 for legume fiber.

Quartiles of overall vegetable intake (g/d) were not associated with breast cancer risk (*P*-trend = 0.2, data not tabulated). Results regarding legume fibers (no association with breast cancer risk) were similar when excluding soya and soya products from the legume food group (data not shown). No interaction between fiber intake and BMI was detected (data not shown).

Sensitivity analyses excluding incident breast cancer cases diagnosed within the first two years of follow-up did not modify the findings (146 cases, out of 4663 included women), nor did sensitivity analyses including only women who completed at least six 24h-dietary records during the first two years of follow-up (cases = 158, out of 3771 included women) or including women who provided at least one 24h-dietary record (cases = 204, out of 5710 included women). We also performed analyses considering dietary fiber intake as a time-dependent variable with one averaged value of intake per year of follow-up (number of included cases = 204, out of 5710 included women). Again, this did not modify our findings (data not shown).

## Discussion

In this prospective study, we observed an inverse association between vegetable fiber intake and breast cancer risk, but no association with total dietary fiber intake or fiber intake from other food sources.

The two recently published meta-analyses [23], [24], as well as the subsequent prospective study on the EPIC cohort [25] observed an inverse association between total dietary fiber and breast cancer risk. However, this association was borderline significant in the EPIC cohort study (P_trend_ = 0.03 but HR_Q5–Q1_ =  0.95 (0.89, 1.01)). The fact that we did not observe any association between total dietary fiber and breast cancer risk in our study may be explained by lack of statistical power, and insufficient contrast between compared quartiles of dietary fiber intake. Indeed, in the recent meta-analysis published by Aune et al. [24], the inverse association between total dietary fiber intake and breast cancer risk was only observed among studies with a large range (>13 g/day) or high level of intake (>25 g/day) in stratified analyses. In our study population, the proportion of women who reached 25 g/day of total dietary fiber was low (only 8.6%).

The meta-analysis of Aune et al. [24] did not detect statistically significant association between fiber intake from different food sources and breast cancer risk. In contrast, our result of an inverse association between vegetable fiber intake and breast cancer risk was consistent with the findings observed in one recent case-control study [36] and in the large recent prospective EPIC study [25], where vegetable fibers were the only fiber subtype associated with decreased breast cancer risk. Additional epidemiological studies including wide ranges of fiber intakes from each food sources and assessing precisely these intakes are needed to more thoroughly elucidate the associations between each type of fiber and breast cancer risk.

In this study, overall vegetable intake (in g/d) was not associated with breast cancer risk, which supports a specific effect of vegetable fiber in breast cancer prevention. In addition, we adjusted for a healthy dietary pattern and for several lifestyle factors (e.g. physical activity, smoking status, etc.). Thus, the inverse association observed in the present study between vegetable fiber intake and breast cancer risk could not be entirely explained by a more general effect of vegetable intake or overall dietary/lifestyle pattern.

Mechanistic data support the plausibility of a protective effect of dietary fiber on breast carcinogenesis, especially vegetable fiber. Vegetable fibers combine insoluble (cellulose) and soluble (pectic substances) fibers [37], [38] in equal proportions. A similar 1:1 combination of soluble and insoluble fibers (psyllium and wheat bran) has been shown to be efficient in the protection against mammary tumorigenesis in rats [39].

The decrease of circulating estrogen concentration by dietary fibers [16], [40] may result at least in part from a modified enterohepatic circulation of estrogens [41], [42], through decrease in the colonic β-D-glucuronidase activity [39], [43], an enzyme allowing estrogens to re-enter the circulation [39], [43], and binding to estrogens, resulting in increased fecal excretion [40], [41]. The influence of dietary fiber on estrogen metabolism may vary according to their biochemical properties (e.g., solubility, fermentability and/or ionic exchange capacity).

Fermentation of dietary fibers in the colon produces SCFA [44], in particular butyrate [45] and propionate [46], which enter the circulation [47] and may exert an anti-inflammatory role [20], [48]. Vegetable fibers provide on average 76% acetate, 14% propionate and 10% butyrate [38] with soluble fibers being highly fermented [40].

Strengths of our study pertained to its prospective design with long follow-up and to the diversity of types and sources of dietary fibers investigated. Moreover, the precise assessment of dietary fiber intake through repeated 24h-dietary records (at least 3, mean = 9.2±3.4) also represents a strength compared to studies that used a food frequency questionnaire (FFQ), which is known to provide a good classification of subjects but a less precise estimation of dietary (and thus fiber) intake and an attenuation of the estimated relative risks [49]. Indeed, a recent prospective study on dietary fiber and colorectal cancer risk compared two dietary assessment tools, i.e., food diaries and FFQ within the same study population and observed statistically significant associations only when dietary fiber was measured by food diaries [50].

However, some limitations should be considered. First, although the number of overall breast cancer cases was reasonably large, it did not allow us to investigate all histological and receptor types of breast cancers (apart from the main subtypes, i.e., postmenopausal, ductal and ER+ or PR+). Nevertheless, even if our ability to detect some of the hypothesized observations may have been limited by the number of cases, this is unlikely to explain the observed relationships which were statistically significant despite the potential power limitation. Second, our results could also have been affected by residual or unmeasured confounding. However, a broad range of common breast cancer risk factors were taken into account (notably dietary, lifestyle, and anthropometric factors). Third, the possibility of chance finding cannot be excluded. However, the number of tests performed in this study is relatively restricted. In addition, we strove to specify our models well, adjusting for the most pertinent covariates, to minimize the potential for Type I error. Moreover, our results are hypothesis driven and supported by epidemiologic literature and biologic plausibility. Thus, the observed findings cannot be explained entirely by chance. Next, caution is needed when extrapolating our results to the whole French female population as this study was based on a sample of volunteers in a cohort study on nutrition and health and were overall better educated and belonged to higher socio-professional categories. However, dietary fiber consumption levels in our study were close to the levels estimated in a national French descriptive survey based on a representative sample [51]. In addition, as the main objective of this study was to investigate the association between individual-level dietary fiber consumption and breast cancer risk, diversity in dietary fiber intake (more than representativeness) was regarded as the important parameter. Besides, in the SU.VI.MAX study, women over 50 underwent a screening mammogram every couple of years during the clinical examination [27]. This regular follow-up increased the chance of diagnosis, in particular at early stage, which is why the mean age at diagnosis was relatively low. Although this systematic testing introduced a difference of diagnosis probability between our population study and the general French population, it also avoided diagnosis bias, which represents a strength of this epidemiological study, since all participants were regularly tested and not only health-conscious women. Finally, no information was available in this study regarding fermentability or ionic exchange capacity of dietary fibers although these parameters would be of interest, in particular when considering the effect of dietary fibers on estrogen metabolism.

In conclusion, this prospective study supports the evidence of an inverse association between vegetable fiber intake and breast cancer risk, in line with experimental data. Further mechanistic studies, large prospective epidemiological cohorts and primary prevention intervention trials are needed to confirm these findings. Similarly, more research is needed regarding the potential effect of dietary fibers on breast cancer survival or recurrence, since few studies have investigated these aspects so far [52].
